# On the Practical Use of Immersive Virtual Reality for Rehabilitation of Intimate Partner Violence Perpetrators in Prison

**DOI:** 10.3389/fpsyg.2022.787483

**Published:** 2022-05-16

**Authors:** Nicolas Barnes, Maria V. Sanchez-Vives, Tania Johnston

**Affiliations:** ^1^Systems Neuroscience, Institute of Biomedical Research August Pi i Sunyer (IDIBAPS), Barcelona, Spain; ^2^General Directorate of Prison Affairs, Department of Justice, Government of Catalonia, Barcelona, Spain; ^3^ICREA, Barcelona, Spain

**Keywords:** virtual reality, intimate partner violence, offenders, violent behavior, rehabilitation, empathy, embodiment, prisons

## Abstract

Virtual reality (VR) allows the user to be immersed in environments in which they can experience situations and social interactions from different perspectives by means of virtual embodiment. In the context of rehabilitation of violent behaviors, a participant could experience a virtual violent confrontation from different perspectives, including that of the victim and bystanders. This approach and other virtual scenes can be used as a useful tool for the rehabilitation of intimate partner violence (IPV) perpetrators, through improvement of their empathic skills or for training in non-violent responses. In this perspective, we revise and discuss the use of this tool in a prison environment for the rehabilitation of IPV perpetrators with a particular focus on practical aspects based on our experience.

## Introduction

Immersive virtual reality (VR) is a powerful tool that allows people to experience different environments and situations in highly controlled and reproducible conditions with ecological validity. When immersed in virtual environments, participants tend to experience the situations depicted as if they are really happening, despite knowing that they are virtual. This engenders “presence” in virtual environments, which has been described as having two components—the illusion of being in a real place (sense of presence or “place illusion”) and the illusion that the scenario is actually occurring (“plausibility illusion”; Slater, 2009). A substantial amount of literature shows that participants exhibit realistic responses to virtual situations in terms of physiological, emotional, cognitive, and even behavioral reactions (for a review on the topic, see Slater and Sanchez-Vives, 2016). For this reason, VR is increasingly being used in psychological therapy (see Cieślik et al., 2020, for a recent systematic review) and has even been used in the field of forensic psychology (see Sygel and Wallinius, 2021 for a recent review) and for work with sexual offenders (Benbouriche et al., 2014). Recent work in our research group has investigated the use of VR in the area of intimate partner violence (IPV), with two main purposes. Firstly, our aim was to reduce key risk factors for perpetration, such as a lack of empathic skills, and ultimately to diminish the risk of recidivism of violent behaviors. Secondly, we aimed to understand the mechanisms underlying the successful use of VR to this end (Seinfeld et al., 2018, 2021).

Intimate partner violence is a complex phenomenon with respect to its conceptualization, prevention, and rehabilitation. Men who have committed crimes of this type can be sentenced to prison, but in many cases, this measure is replaced by the obligation to attend treatment programs outside of prison, as an alternative criminal measure (probation). In prison, they also have the possibility of undergoing rehabilitation programs, but this is done in a peculiar daily-life environment of deprivation of liberty. Indeed, inmates must live with a series of regulations and situations that are not always fully compatible with rehabilitation activities, such as rigid schedules, regimental regulations, or even the peer pressure to which they may be subjected.

In the Catalan prison system, the main risk factors for perpetration of IPV are evaluated and described with a risk assessment tool named RISCANVI, which is based on the scientific literature on the topic (Andrés-Pueyo et al., 2010). Using RISCANVI, the professionals in the rehabilitation teams evaluate risk factors, such as the presence of mental disorders, reduced cognitive abilities, a lack of family and social support, a history of violence, and poor impulse control and hostility, all of these factors being related to levels of empathy toward the victim.

Although the definition of empathy is somewhat controversial, a largely adopted conceptualization is that of Davis (1980), who distinguishes two components of empathy: an affective component, which is related to the ability to experience others’ emotional states and a cognitive component, which is related to the ability to imagine and understand others mental processes (e.g., the ability to take the perspective of others). A general problem in this area is that the link between empathic skills and violent behavior in general, and IPV in particular, is yet to be clarified, as posed by Jolliffe and Farrington (2004) in their meta-analytic study, and its ulterior replication and extension by Van Langen et al. (2014). This difficulty is due to the fact that empathy is a multidimensional construct that is difficult to conceptualize and measure. Additionally, it is likely that factors such as age and personality affect empathic skills, while empathy, although not always a direct predictor of violence can be a moderator of the link between a risk factor and actual perpetration. In fact, not all components of empathy seem to be affected in criminal offenders and emotion recognition is most consistently found to have an effect (Seidel et al., 2013; Mariano et al., 2017): antisocial behavior (Marsh and Blair, 2008), violent offending (Kret and De Gelder, 2013), and IPV (Marshall, 2005; Babcock et al., 2008; Marshall and Holtzworth-Munroe, 2010) have indeed been related to deficits in emotion recognition. This is perhaps one of the reasons why most perpetrator programs target, among other risk or protective factors, improvement of empathic capacities (Jolliffe and Farrington, 2007; Day et al., 2010; Mann and Barnett, 2013).

While rehabilitation programs often differ between countries, regions, or institutions, they are usually similar in that they are dispensed in a group format, and combine psychoeducational models such as the Duluh model (Pence and Paymar, 1993), with CBT approaches (Gondolf, 2002; Babcock et al., 2004; Feder and Wilson, 2005; Murphy and Meis, 2008; Eckhardt et al., 2013; Karakurt et al., 2019) that focus on reducing risk factors, such as cognitive distortions, skill deficits, and other criminogenic needs (Wexler, 2020).

Coming back to the concept of empathy, at a more clinical level, one of the aims of VR with violent men is to make them aware of their active role in the use of violence: bringing them closer to the sensations and emotions that the victim may feel can help them to have an implicit and explicit understanding of the impact and consequences of their behavior, and improve their empathy and motivation for change (Carbajosa et al., 2017; European Network for Work with Perpetrators, 2018). Without VR, this work is usually achieved through group discussion, role playing, and showing videos depicting IPV scenes or survivor testimonies (e.g., Day et al., 2010).

Although to this day, rehabilitation programs have been found to be somewhat efficacious (Polaschek and Collie, 2004; Jolliffe and Farrington, 2007), results in this regard are ambiguous (Stover et al., 2009; Eckhardt et al., 2013), and effect sizes vary between studies (Babcock et al., 2004; Feder and Wilson, 2005; Miller and Iovanni, 2013). As for the question of empathy, this could be due to the way efficacy is measured: in the field of IPV rehabilitation, it is either measured through recidivism rates (Gondolf, 2004; Stover et al., 2009), or through more clinical measures, focusing on reducing specific risk factors (or deficits) of perpetration. However, this disparity in findings is noted between studies that measured efficacy related to the reduction of recidivism (Gondolf and White, 2001; Shorey et al., 2012; Radatz and Wright, 2016), or to reducing specific risk factors (e.g., lack of empathy; Day et al., 2010; Mann and Barnett, 2013, aggression, and anger; Blacker et al., 2008; Hornsveld et al., 2008).

Our work with perpetrators focuses on the use of VR to improve risk factors for perpetration in men who commit IPV crimes. However, ongoing studies are also measuring the relationship between the inclusion of VR in programs and the decrease in recidivism. The work we report here focusses on empathy as a risk factor, in its more cognitive aspect (through embodied perspective taking) and of emotion recognition, measured through a cognitive task (described below) as an outcome of this perspective-taking experience.

According to criminological research, effectiveness of rehabilitation programs improves if they meet certain characteristics, such as relying on a solid conceptual model, being adequately structured (in terms of content, duration, and intensity) to match the criminogenic necessities of participants, and incorporating different treatment techniques (Echeburúa and Amor, 2010; European Network for Work with Perpetrators, 2018). To this end, in recent years, significant effort has been invested in improving rehabilitation programs, both in probation and in prison, by trying to individualize them as much as possible through adapting them to the needs of each individual (Jovanovich, 2019; Lila et al., 2019).

**Figure 1:**
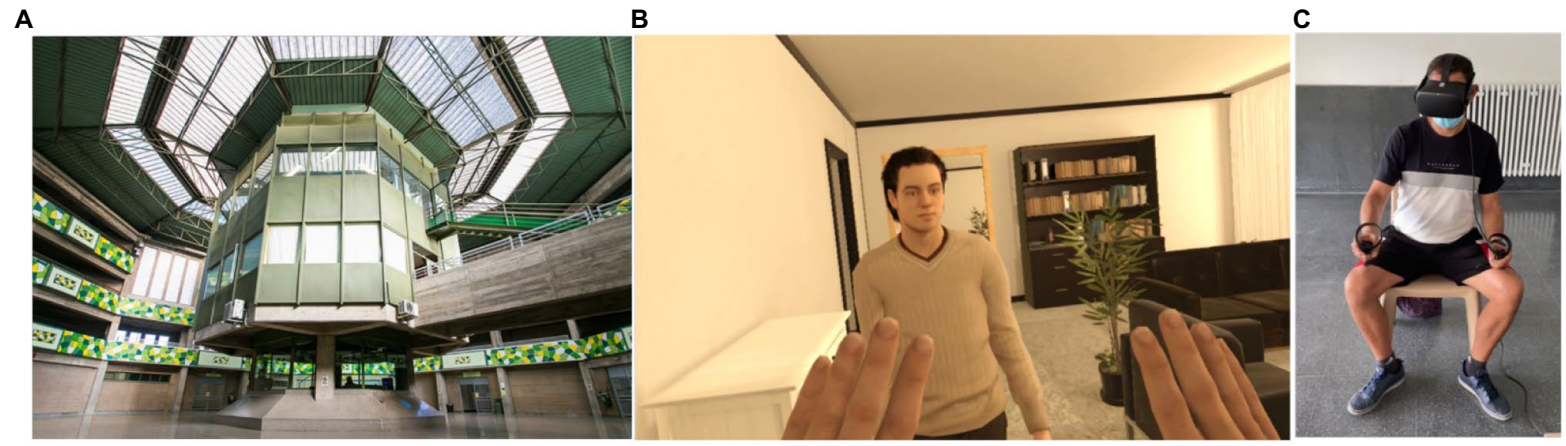
**(A)** Quatre Camins Prison. **(B)** Screenshot of the virtual reality (VR) scene. **(C)** An inmate immersed in the virtual reality scene used in Seinfeld et al. (2018).

In our work with IPV perpetrators in prison (Figure 1) and on probation during the last decade in Catalonia, we found that perpetrators are often sentenced to short-term sentences, inducing us to design interventions and adopt methodologies that are time-efficient and directly geared to their specific criminogenic needs. For this reason, interventions for rehabilitation can benefit from innovative methodologies such as immersive VR that can offer IPV offenders an immersive, experiential, and effective learning process through direct exposure to different types of scenarios, and through the experience of new perspectives.

A founding study using VR in the field of IPV was conducted with a sample of men convicted to a period of probation and rehabilitation for IPV offences (Seinfeld et al., 2018). Participants were immersed in a virtual scene (Figure 1B) in which they were embodied as a woman experiencing IPV from her male partner (i.e., they were virtually placed in her body and experienced the scene from her perspective). Emotion recognition skills were measured before and after exposure to this scene, with the Face-Body Compound task (Meeren et al., 2005), a cognitive task in which participants must classify facial expressions. The results showed that these perpetrators significantly improved their emotion recognition skills, which were lower at baseline compared with a sample of men with no history of violence (Seidel et al., 2013; Seinfeld et al., 2018), after embodiment as the victim of IPV abuse. Following this work, other related studies from our group (Gonzalez-Liencres et al., 2020; Johnston, 2021) have demonstrated that immersive VR is a potentially effective tool for producing neuro-psychological, emotional, and attitudinal changes in IPV perpetrators.

### Focus of the Current Paper

In this article, we draw on our experience investigating the use of immersive VR in different prisons and rehabilitation settings (Figure 1C), to highlight the usability of such a tool for managing risk factors in IPV perpetrators, and to consider how to best integrate immersive VR sessions into their traditional treatment programs. We mainly focus on practical considerations, directly derived from our experience, and the opportunities and challenges of importing this powerful tool into prisons. Some of this research has been carried out in the context of the European research project: VR per Genere (Virtual Reality Prevention of Gender Violence in Europe based on Neuroscience of Embodiment, PeRspective, and Empathy; www.vrpergenere.com), which aims to use the advantages of VR for the prevention of IPV and for the rehabilitation of perpetrators through the improvement of classic rehabilitation strategies outlined previously. As will be developed below, our work proposes VR as a promising method for improving empathy and reducing specific risk factors for perpetration in offenders (Seinfeld et al., 2018; Barnes, 2020; Johnston, 2021) and as a means for individualizing treatment to participants’ specific needs in a cost-effective manner, through the presentation of different scenarios. The VR per Genere project also deals with using VR tools for the prevention of gender violent behaviors targeting younger populations.

The current paper will group practical considerations into three themes: (1) considerations related to the individual differences between offenders; (2) considerations related to the factors that may affect the response of the offenders when talking about their VR experience; and (3) considerations related to the prison environment in which immersive VR is applied. This specific segmentation is operational, but these three themes are highly interrelated.

## Individual Differences of Offenders in Prison

In our recent work with VR prison settings with the aim of rehabilitation of IPV offenders (see Figure 1); we have identified several individual differences that should be accounted for when designing treatment programs. We will discuss these in turn.

### Cognitive Skills

In our studies, we have assessed different psychological and empathic outcomes in order to understand whether these factors might be improved by *ad hoc* virtual environments (e.g., Seinfeld et al., 2018; Johnston, 2021). To this end, different tests have been administered in a questionnaire format, such as the Interpersonal Reactivity Index (IRI; Davis, 1980), a measure of empathy; the social desirability questionnaire (Chico and Ferrando, 2000), or the Autonomic Perceptions Questionnaire (APQ; Mandler et al., 1958), a measure of perceived internal states. We have found that, on the whole, our participants had difficulty understanding these psychometric tests, whether because of their language and/or reading skills (e.g., they did not understand some words, or the whole sentence), abstraction skills (e.g., they did not understand the concept of a Likert scale, which evaluates the degree of agreement with a statement, based on a numerical scale), or concentration skills (e.g., they had difficulties focusing their attention long enough to complete the questionnaire). For this reason, with many participants, the researchers were required to read the whole questionnaire item by item, to ensure those participants’ answers were issued based on a correct understanding of the questions. This suggests it is necessary to adapt the evaluation of some psychological constructs to the characteristics of the penitentiary population, in research as well as in applied contexts.

Our experience suggests that other types of evaluation techniques might be recommended for this population, such as behavioral, indirect, or implicit measures: emotion recognition tests, which rely on cognitive tasks that to a certain extent are not confounded by abstraction or the language skills of the participants; semi-structured interviews, which rely on verbal rather than written communication; or questionnaires with simple questions that rely less on abstract concepts. Another option, which can be facilitated by the use of VR, is to carry out behavioral evaluations: because people tend to respond realistically when immersed in VR (Slater and Sanchez-Vives, 2016), observing participants’ reactions to (virtual) events (e.g., Rovira et al., 2009; Slater et al., 2013, in the field of violence, and Seinfeld et al., 2018; Johnston, 2021, in the field of IPV), can allow us to tap into different psychological constructs in order to overcome cognitive skills deficit, as well as the problem of social desirability, which will be discussed below. In short, assessment options that are less influenced by the understanding of written language and by intellectual skills might be preferable when working with penitentiary populations.

### Social Desirability Levels

Previous studies have reported higher social desirability in forensic evaluation contexts in imprisoned populations (Sanz Fernández et al., 2018), as well as in offenders on probation (Seinfeld et al., 2018; Johnston, 2021). However, we have not yet found that this is a factor that could compromise the *outcome* evaluation processes in prison samples. In a recent study of offenders in prison, we detected social desirability scores that were similar to non-offending populations and these scores did not influence empathy questionnaires (Barnes, 2020). Although in classical clinical and forensic contexts, one can expect response tendencies from individuals to show the best version of oneself to the researcher or examiner (Henning et al., 2005; Eckhardt et al., 2012), in the penitentiary context this is not always the case, at least once the person has already begun their personalized rehabilitation itinerary. In a recent study conducted in a prison population, social desirability levels were similar to those of the general population and did not affect participants’ responses to questionnaires, contrary to our previous studies. This suggests that participants in prison might not respond in a defensive way, or in what they might perceive as a desirable way—relative to prison social norms—when asked how they *felt* during a particular VR experience, which might impede the evaluation process. In agreement with this, some authors have found that impression management may be an enduring individual characteristic within an offender sample rather than a situationally determined response (Mills et al., 2003).

### Criminal Profiles of the IPV Perpetrators

Formal experiments as well as informal comparisons of results obtained in prison vs. probation population samples highlight the differences coming from the criminal profiles of the IPV perpetrators in two aspects: (1) the outcomes of evaluations of some psychological constructs, and (2) the effectiveness of the immersive VR intervention.

It is a consistent finding in the literature that emotion recognition is lower in violent offenders (Seidel et al., 2013). This was the case in IPV probation offenders compared with non-violent controls (Seinfeld et al., 2018). Our preliminary results from prison perpetrator samples on the same emotion recognition task as that used by Seinfeld et al. (2018) are that emotion recognition in this population is in turn worse than those of probation samples (Johnston, 2021). Furthermore, within the prison population itself, a recent investigation (Barnes, 2020) pointed toward the idea that different criminal profiles (of more or less severity, measured in terms of the number of committed crimes and the severity of crimes) may benefit differently from a single-session VR intervention: results obtained on an empathy questionnaire (IRI), after embodiment as the victim, suggest that prisoners with a less severe or lower risk profile present greater improvements in empathy when compared with those with more severe profiles. In fact, although we have measured criminal profiles based on the previously described criteria, many other psychological and behavioral factors should also be taken into account in order to fully assess this dimension. This could not be included in our studies for practical research reasons (e.g., length of experiments and number of variables); however, future studies will try to include them. For instance, the social desirability tendencies we highlighted earlier might also be related to criminogenic profiles in the sense they could be correlated with personality traits, such as narcissism, Machiavellianism, and psychopathy (Kowalski et al., 2018). We would like to argue that factors such as personality, (e.g., callousness of affects or psychopathic traits), dispositional traits (e.g., disposition to anger, alexithymia; Birkley and Eckhardt, 2015; Gillespie et al., 2018), attitudinal traits (e.g., perceptions relative to violence; Eckhardt et al., 2012), or comorbidities (e.g., addiction and trauma) likely also need to be studied as contributors to the response to VR and as components of criminal profiles (Babcock et al., 2008).

In short, psychologists and educators from the prisons that we work with (CP Mas d’Enric and CP Quatre Camins) have suggested the need to have virtual situations with greater emotional load that can be better adapted to the different IPV perpetrator criminal profiles. In this sense, there is a consideration to be made: to what extent should the contents of the situations in VR for rehabilitation of IPV offenders be adapted to their personal profile and level of violent behavior? To answer these highlighted needs, in a recent study, we explored the possibility of personalizing the VR experience to the perpetrators’ needs, through intensification of the VR experience by adding fake interoceptive feedback that was representative of fear (i.e., they heard an accelerated heartbeat and felt a vibration on the chest that was synchronized to the sound) whilst participants experienced the scene (Johnston, 2021). Although these changes did not result in significant changes in outcomes after the VR experience, prisoners with longer sentences (usually related to severity of crime, to profiles of greater risk, or to a greater number of committed crimes) were found to report higher interoceptive feelings inside VR, suggesting different experiences of the same VR scenes based on their profiles. To summarize, we could say that the same virtual intervention does not work in the same way for all individuals, and ongoing research should aim to disentangle the finer points of this observation.

### Psychological Adjustment

Some offenders that initially planned to attend the VR intervention session were discarded from our studies on the day of the session due to momentary psychological maladjustment or to high levels of stress, as evaluated by a forensic psychologist. Being in a prison, despite recent significant advances in the humanization of prison environments, can inevitably affect a person socially, psychologically, and even biologically, with high levels of stress, periods of adaptation, repercussions at the family level, and so on, all of which can affect physical health (Pereira et al., 2016). Additionally, several offenders also suffered from pre-existing mental disorders, which can be exacerbated by the prison context.

This raises a consideration: while any rehabilitation process can be emotionally costly for participants, immersive VR has the power to elicit strong emotional responses (Diemer et al., 2015). This has the advantage of allowing experiential learning and can elicit significant psychotherapeutical change (Cieślik et al., 2020). For this reason, it is necessary—as with any therapeutic tool—to assess when the optimal moment for participants to be the most receptive to the VR intervention might be. It is also necessary to be attentive to the situational psychological state of each participant both before and during the intervention. Hence, rehabilitation clinicians, who evaluate implicitly the fitness of each member for their group intervention, must extend this judgement to participants’ momentary disposition in order to benefit maximally from a VR session.

Additionally, to avoid any exposure to experiences that might result in psychological decompensation, clinicians should be particularly attentive to discard subjects with pre-existing psychological disorders that affect perceptions of reality, such as psychosis. Indeed, while some VR scenes have been successfully tailored specifically to such disorders (using for instance the power of VR to work on paranoia; Veling et al., 2014), the therapeutic work that takes place during the VR scenes we have described thus far, aimed at rehabilitation of IPV, is not adapted to the specific needs of this population.

In sum, as for any therapeutic tool, the VR intervention should ideally be integrated into a rehabilitation program with an adequate follow-up, not only to evaluate the state of the person, but also to help them reflect upon their experience in order to obtain maximum benefit from it. This point will also be discussed in the Ethical Considerations section below.

## Factors That Affect Perpetrators’ Responses When Talking About Their VR Experience

In the previous sections, we considered social desirability and the little impact that this phenomenon seems to have on prison participants’ responses to empathy questionnaires. But is it possible that social desirability or other social factors affect how perpetrators in prison report their *experience* of the VR sessions? We believe that important attitudinal and emotional factors—inherent to the social and clinical context of IPV perpetrators and of the prison itself—likely come into play in how imprisoned perpetrators talk about their experience inside VR and in how they might experience the treatment or rehabilitation process itself.

One of the common characteristics of IPV perpetrators is the influence that gender stereotypes and hegemonic masculinity values (e.g., “boys do not cry” and “real men must force respect”), cognitive distortions (e.g., “all women are the same” and “the law is always in favor of women”), and cognitive deficits (e.g., alexithymia, deficits in emotion recognition of others) have on their way of relating to their environment and their way of managing emotions (Echeburúa and Amor, 2010). In addition to these individual characteristics, the prison context itself inherently carries an extra layer of these social and gendered norms (see for instance Jewkes, 2005; Michalski, 2017). This might influence not only participants’ evaluations of the situations depicted in the scenes, but also what they say when talking about their immersive VR experience, whether during evaluations or in clinical interviews. This is particularly pertinent if the person (researcher or clinician) evaluating the outcome of the VR has not built a therapeutic alliance with them. It is then likely that they apply cognitive control over their answers in order to fit with prison norms. This behavior might not be intended to present a desirable profile to pass an evaluation, or to obtain benefits, as we would usually define social desirability in a forensic context (e.g., faking an aversion for violence, or empathy for the victim in questionnaires). However, this could still be considered a form of socially desirable responding in the sense that perpetrators might depict an image of themselves that they consider desirable in the prison environment. For instance, they could report they did not feel scared while embodied as the woman because they fear appearing weak, or because they do not consider that talking about emotions is something suitable for a prison environment. Therefore, under this hypothesis, when evaluating prison perpetrators genuine experiences in VR, the medium through which this evaluation is carried out will be important. Here again, evaluation measures that are more implicit or indirect should be preferred. For example, in the case of evaluation of participants’ emotional experience, explicit evaluation could be more apparently related to physical sensations rather than emotions *per se* (e.g., through the use of the Autonomic Perception Questionnaire), which could then lead to a conversation about their experience inside VR with the clinician. Another solution is to *observe* participants’ behavioral and physiological responses (e.g., heart rate and skin conductance) inside VR. Indeed, participants are known to respond realistically when immersed in VR (Slater and Sanchez-Vives, 2016) and a recent study conducted with IPV perpetrators in probation (Johnston, 2021) showed that although they responded in a highly socially desirable way on explicit questionnaires related to acceptance of violence, they responded realistically (i.e., in a way that demonstrated acceptance of violence) inside VR when confronted with a situation of violence experienced as a bystander.

## Influence of Contextual Factors on Personal Experience Inside VR

No one will be surprised if we affirm that the prison environment and serving a prison sentence is not a situation comparable to life outside prison, due to the inherent restrictions of freedom and institutionalized control prisoners experience. In addition, it can cause considerable levels of stress on the individual (Kołodziej et al., 2021). An interesting question is whether this can affect the effectiveness of immersive VR sessions.

Among other factors that might influence empathic responses, various studies point toward the idea that stress plays a role. In a study conducted in VR (Flasbeck et al., 2018), excessive stress levels were found to attenuate empathic responses to the pain of another person, while mild stress intensified this empathic response. In perpetrator populations, baseline empathic skills are known to be low (Jolliffe and Farrington, 2004; Van Langen et al., 2014) and it is possible that this prison environment reduces their general empathic skills even further. The studies we have conducted (Barnes, 2020; Johnston, 2021) suggest that participants in prison do manage, despite the stressful situation, to experience the key illusions necessary for any VR experience (e.g., Slater and Sanchez-Vives, 2016) and benefit from the experience of VR. Regarding the notion of criminal profiles, in one study, participants with lower criminogenic needs and lower number of crimes improved their empathy (Barnes, 2020)and in another study they improved some aspects of emotion recognition (Johnston, 2021).

Prisonization, building upon the question of social norms in the prison context, is another factor that affects responses when talking about their experience of VR, together with other processes related to adjustment to prison. Criminals in prison tend to adopt a defensive attitude as a defense mechanism against a hostile environment. According to some studies (Pereira et al., 2016), it seems that when facing problems, prison populations prefer avoidance strategies over the use of coping strategies of logical analysis or seeking support. Prisonization could be defined as the process in which the aggressor incorporates new rules in his life habits, in his way of thinking, feeling, and expressing those feelings, in addition to his way of acting (e.g., Pereira et al., 2016). As elaborated previously, the prisonization process may influence the explicit responses to the VR session evaluations, which calls into question the reliability of these responses. For instance, participants might be compelled to respond in a way that they perceive would fit the stereotype of a strong man (e.g., report they did not feel scared or vulnerable inside the VR, in an attempt to maintain their self-perceived manly image). Again, one of our proposals would be to include more implicit measures that could be less influenced by culture and language, and could hence overcome the socially desirable response participants tend to make, related to their social norms associated with prisonization. On the other hand, perhaps future adaptations of immersive VR scenes could help to modify these social norms related to the effects of prisonization. Following work by Gonzalez-Liencres et al. (2020) and Johnston (2021), which showed that embodiment as the victim brought about changes in key attitudes toward women and victims, future work could target the sets of social norms related to the effects of prisonization.

Another difficulty lies in what we could call the “contextual distraction” of the prison environment. For a VR immersion to be experienced as real, several conditions must be met. Here it is necessary to talk about the virtual illusions that lead participants to feel and behave as if they really were inside the VR scene they are experiencing (Slater, 2009), namely the place illusion (the sensation of being in the place depicted inside VR instead of the real world), and the plausibility illusion (the feeling that what is being experienced inside VR is really happening). Sources of contextual distraction from the real world such as external noises or physical objects that are perceived inside VR (e.g., a chair and a wall) can result in a break in the sense of presence; hence, it is important to maintain a quiet and spacious environment when using VR. In our experience, the prison environment is not always favorable to this objective, either due to ambient noise (e.g., the public address system may disrupt the VR experience) or to distractions related to institutional life (e.g., a guard enters the room during the VR experience, or the perpetrator is called by the guards for an activity or appointment). Additionally, although this was not found to influence participants’ experience *inside* VR, they often arrive to the session with a level of distraction activated by institutional life (e.g., a discussion that they may have recently had with another inmate, the obligations of the day, and restrictions on speaking with loved ones). In other words, participants might not feel high levels of key illusions because of these distractors: this in turn could influence the efficacy of any VR intervention. In short, factors relative to the process of immersion and presence have to be borne in mind, and where possible protected by the facilitators of the VR therapeutic process by, for example, choosing the most appropriate space, reinforcing coordination with the guards and explaining to them the importance of the process, having a short talk with the participant before starting the process, in order to help them stay “in the moment,” and so on.

## Ethical Considerations

Before we conclude, we should highlight some ethical considerations. Since simulation of real life situations that could bring about intense negative emotions in participants has been highlighted as potentially unethical (Madary and Metzinger, 2016), VR has increasingly been suggested as a substitute for real-life experiments that are ecologically valid yet experimentally controlled (Parsons, 2015). Through the illusions of presence and plausibility (Sanchez-Vives and Slater, 2005; Slater, 2009), virtual reality has indeed proven to be a useful tool to simulate and study social problems, such as violence, in a way that overcomes many obstacles to research, such as safety issues, for instance.

However, we would like to emphasize that the development and use of any VR tool must be carefully considered from an ethical perspective (Aymerich-Franch and Fosch-Villaronga, 2020; Marloth et al., 2020; Slater et al., 2020). According to the British Psychological Society, the main ethical considerations when conducting research (which can also be applied to psychological interventions) include the intended value of the research, the respect of dignity and autonomy of the participants, the general social responsibility of the research, and the maximization of benefit and minimization of harm (BPS, 2014).

Applied to the topic we are treating here, in order to ensure that we respect the dignity and autonomy of the participants and that the potential benefit/harm ratio is acceptable, a careful forensic and clinical evaluation is necessary prior to using immersive VR tools. What psychological impact might being in the perspective and the body of the victim have? Has the participant experienced situations of domestic violence as a child? If so, will this experience be more harmful than beneficial for them or their rehabilitation path? How will the participant’s negative emotional experience inside VR translate into positive therapeutic change? These are some of the questions that must be addressed by the clinicians in charge of the program. Indeed, interventions should take into consideration the individual needs of each participant in order to maintain a balance between the necessity to tackle the problematic issues related to violence (as with any rehabilitation strategy) and protecting the emotional and psychological integrity of each participant. In a nutshell, any VR intervention or study in this population should be part of a carefully planned rehabilitation strategy, where the emotional and psychological impact must be carefully and regularly monitored.

For this reason, our VR studies have always been carried out as part of the perpetrators’ traditional rehabilitation programs, with various group and individual sessions planned before and after VR. Further, our VR interventions are always integrated into the objectives of the existing rehabilitation program, held out in probation and in prison in Catalonia. This enables us to not only control participants’ understanding of the scene, but also permits the potential elaboration of the emotional impact the scene might have and to avoid a harmful experience.

## Conclusion

State-of-the-art research in immersive VR offers numerous possibilities for developing tools that can be integrated into rehabilitation programs in prisons and probation. In particular, here we have discussed those that use virtual embodiment in order to take someone else’s perspective, which can be valuable for enhancing empathic behavior and reducing violence. Further, we have discussed how virtual environments can also be used as a tool to evaluate tolerance of violent behavior. It is important to carry out basic research to determine how to best use these technologies as well as understanding the mechanisms involved and their neuroscientific basis. VR can be a useful tool as an integral part of rehabilitation programs for IPV offenders for several reasons, such as the implicit learning that it allows and the improvement of empathic skills, but also the evaluation of behavioral reactions when confronted with violence, the development of healthy social norms, and for increasing the level of motivation in criminal populations, which can be particularly demotivated toward rehabilitation (Ticknor and Tillinghast, 2011).

However, when we take these tools from the laboratory to a prison, many factors should be taken into account, since the conditions in this context are complex and can be affected by various interferences. This is important both if we use these tools in prison for research or for rehabilitation. Here, we have discussed some of these factors ranging from cognitive skills, to stress levels associated with imprisonment or social desirability, and their consideration stems from our practical experience.

Although VR will not solve the problem of gender violence by itself, there is no doubt that it can become an additional tool in the rehabilitation of criminal behavior. In fact, VR could even serve as a tool for the reintegration of people back into society (Ticknor, 2018), and will benefit from the increasing scientific evidence for its effectiveness in various areas of psychological health. VR can be utilized for rehabilitation, prevention, and awareness through the modification of behaviors and attitudes related to violence and can be integrated, in a relatively simple way, into traditional rehabilitation programs.

## Data Availability Statement

The original contributions presented in the study are included in the article/supplementary material; further inquiries can be directed to the corresponding author.

## Ethics Statement

The individual(s) provided their written informed consent for the publication of any identifiable images or data presented in this article.

## Author Contributions

All authors listed have made a substantial, direct and intellectual contribution to the work, and approved it for publication.

## Funding

The research related in this perspective paper was funded by the European Union’s Rights, Equality and Citizenship Program (2014–2020) under Grant Agreement: 881712 (VRperGenere).

## Conflict of Interest

MVS-V is one of the founders of Virtual Bodyworks Inc.

The remaining authors declare that the research was conducted in the absence of any commercial or financial relationships that could be construed as a potential conflict of interest.

## Publisher’s Note

All claims expressed in this article are solely those of the authors and do not necessarily represent those of their affiliated organizations, or those of the publisher, the editors and the reviewers. Any product that may be evaluated in this article, or claim that may be made by its manufacturer, is not guaranteed or endorsed by the publisher.

## References

[ref1] Andrés-Pueyo A. Arbach-Lucioni K.I. Redondo S. (2010). Informe RisCanvi. Memoria técnica de la construcción del protocolo y las escalas de valoración del riesgo de violencia para delincuentes violentos (RisCanvi-S, RisCanvi-C y E-Riscanvi). Volumen primero. Informe técnico. Generalitat de Catalonia.

[ref2] Aymerich-Franch L. Fosch-Villaronga E. (2020). A self-guiding tool to conduct research with embodiment technologies responsibly. Front. Robot. AI 7:22. doi: 10.3389/frobt.2020.00022, PMID: 33501191 PMC7805620

[ref3] Babcock J. Green C. Robie C. (2004). Does batterers’ treatment work? A meta-analytic review of domestic violence treatment. Clin. Psychol. Rev. 23, 1023–1053. doi: 10.1016/j.cpr.2002.07.001, PMID: 14729422

[ref4] Babcock J. Green C. Webb S. (2008). Decoding deficits of different types of batterers during presentation of facial affect slides. J. Fam. Violence 23, 295–302. doi: 10.1007/s10896-008-9151-1

[ref5] Barnes N. (2020). El proyecto V-Respect.Me en el Programa de violencia de género en los centros penitenciarios

[ref6] Benbouriche M. Nolet K. Trottier D. Renaud P. (2014). "Virtual reality applications in forensic psychiatry.” in *Proceedings of 2014 Virtual Reality International Conference VRIC*; April 14, 2014; Laval, France, 1–4.

[ref7] Birkley E. L. Eckhardt C. I. (2015). Anger, hostility, internalizing negative emotions, and intimate partner violence perpetration: a meta-analytic review. Clin. Psychol. Rev. 37, 40–56. doi: 10.1016/j.cpr.2015.01.002, PMID: 25752947 PMC4385442

[ref8] Blacker J. Watson A. Beech A. R. (2008). A combined drama-based and CBT approach to working with self-reported anger aggression. Crim. Behav. Ment. Health 18, 129–137. doi: 10.1002/cbm.686, PMID: 18383198

[ref9] BPS (2014). British psychological society code of human research ethics.

[ref340] Carbajosa P. Catalá-Miñana A. Lila M. Gracia E. Boira S. (2017). Responsive versus treatment-resistant perpetrators in batterer intervention programs: Personal characteristics and stages of change. Psychiatry, Psychology and Law 24, 936–950., PMID: 31984001 10.1080/13218719.2017.1347933 PMC6818265

[ref10] Chico E. Ferrando P. J. (2000). Adaptación y análisis psicométrico de la escala de deseabilidad social de marlowe y crowne. Psicothema 12, 383–389.

[ref11] Cieślik B. Mazurek J. Rutkowski S. Kiper P. Turolla A. Szczepańska-Gieracha J. (2020). Virtual reality in psychiatric disorders: a systematic review of reviews. Complement. Ther. Med. 52:102480. doi: 10.1016/j.ctim.2020.102480, PMID: 32951730

[ref12] Davis M. H. (1980). A multidimensional approach to individual differences in empathy. JSAS Cat. Sel. Doc. Psychol. 10:85

[ref13] Day A. Casey S. Gerace A. (2010). Interventions to improve empathy awareness in sexual and violent offenders: conceptual, empirical, and clinical issues. Aggress. Violent Behav. 15, 201–208. doi: 10.1016/j.avb.2009.12.003

[ref14] Diemer J. Alpers G. W. Peperkorn H. M. Shiban Y. Mühlberger A. (2015). The impact of perception and presence on emotional reactions: a review of research in virtual reality. Front. Psychol. 6:26. doi: 10.3389/fpsyg.2015.00026, PMID: 25688218 PMC4311610

[ref15] Echeburúa E. Amor P. J. (2010). Perfil psicopatológico e intervención terapéutica con los agresores contra la pareja. Rev. Española Med. Leg. 36, 117–121. doi: 10.1016/S0377-4732(10)70040-7

[ref16] Eckhardt C. I. Murphy C. M. Whitaker D. J. Sprunger J. Dykstra R. Woodard K. (2013). The effectiveness of intervention programs for perpetrators and victims of intimate partner violence. Partn. Abus. 4, 1–26. doi: 10.1891/1946-6560.4.2.e17, PMID: 32886666

[ref17] Eckhardt C. I. Samper R. Suhr L. Holtzworth-Munroe A. (2012). Implicit attitudes toward violence among male perpetrators of intimate partner violence: a preliminary investigation. J. Interpers. Violence 27, 471–491. doi: 10.1177/0886260511421677, PMID: 22333320

[ref18] European Network for Work with Perpetrators (2018). Guidelines to develop standards for programmes working with perpetrators of domestic violence, Version 3.

[ref19] Feder L. Wilson D. B. (2005). A meta-analytic review of court-mandated batterer intervention programs: can courts affect abusers’ behavior? J. Exp. Criminol. 1, 239–262. doi: 10.1007/s11292-005-1179-0

[ref20] Flasbeck V. Gonzalez-Liencres C. Brüne M. (2018). “The brain that feels into others: Toward a neuroscience of empathy,” in The Neuroscience of Empathy, Compassion, and Self-Compassion. eds. Stevens L. Woodruff C. C. (New York: Elsevier Academic Press).

[ref21] Gillespie S. M. Garofalo C. Velotti P. (2018). Emotion regulation, mindfulness, and alexithymia: specific or general impairments in sexual, violent, and homicide offenders? J. Crime Justice 58, 56–66. doi: 10.1016/j.jcrimjus.2018.07.006, PMID: 33404621

[ref22] Gondolf E. W. (2002). Batterer Intervention Systems: Issues, Outcomes, and Recommendations. Thousand Oaks: SAGE Publications, Inc.

[ref23] Gondolf E. W. (2004). Evaluating batterer counseling programs: a difficult task showing some effects and implications. Aggress. Violent Behav. 9, 605–631. doi: 10.1016/j.avb.2003.06.001

[ref24] Gondolf E. W. White R. J. (2001). Batterer program participants who repeatedly reassault. J. Interpers. Violence 16, 361–380. doi: 10.1177/088626001016004006

[ref25] Gonzalez-Liencres C. Zapata L. Iruretagoyena G. Seinfeld S. Pérez-Mendez L. Arroyo-Palacios J.. (2020). Being the victim of intimate partner violence in virtual reality: first- versus third-person perspective. Front. Psychol. 11:820. doi: 10.3389/fpsyg.2020.00820, PMID: 32457681 PMC7225265

[ref26] Henning K. Jones A. R. Holdford R. (2005). “I didn’t do it, but if I did I had a good reason”: minimization, denial, and attributions of blame among male and female domestic violence offenders. J. Fam. Violence 20, 131–139. doi: 10.1007/s10896-005-3647-8

[ref28] Hornsveld R. H. J. Nijman H. L. I. Kraaimaat F. W. (2008). Aggression control therapy for violent forensic psychiatric patients: first results. Psychol. Crime Law 14, 1–18. doi: 10.1080/10683160701340569, PMID: 17636205

[ref29] Jewkes Y. (2005). Men behind bars: “doing” masculinity as an adaptation to imprisonment. Men Masculinities 8, 44–63. doi: 10.1177/1097184X03257452

[ref30] Johnston T. (2021). Assessment, prevention and rehabilitation of intimate partner violence through immersion in virtual reality. Modifying Cognitions, Emotions and Behaviours through Embodied Perspective Taking. Doctoral dissertation. University of Barcelona, Spain.

[ref31] Jolliffe D. Farrington D. P. (2004). Empathy and offending: a systematic review and meta-analysis. Aggress. Violent Behav. 9, 441–476. doi: 10.1016/j.avb.2003.03.001

[ref32] Jolliffe D. Farrington D. P. (2007). A systematic review of the national and international evidence on the effectiveness of interventions with violent offenders. Great Britain.

[ref33] Jovanovich S. (2019). Probation and prison based programmes for perpetrators of domestic and sexual violence: a European overview.

[ref34] Karakurt G. Koç E. Çetinsaya E. E. Ayluçtarhan Z. Bolen S. (2019). Meta-analysis and systematic review for the treatment of perpetrators of intimate partner violence. Neurosci. Biobehav. Rev. 105, 220–230. doi: 10.1016/j.neubiorev.2019.08.006, PMID: 31415863 PMC6742529

[ref35] Kołodziej K. Kurowska A. Majda A. (2021). Intensity of perceived stress and control of anger, anxiety and depression of people staying in polish penitentiary institutions. Int. J. Prison. Health doi:10.1108/IJPH-12-2020-0103 [Epub ahead of print]. 34390549

[ref36] Kowalski C. M. Rogoza R. Vernon P. A. Schermer J. A. (2018). The dark triad and the self-presentation variables of socially desirable responding and self-monitoring. Pers. Individ. Differ. 120, 234–237. doi: 10.1016/j.paid.2017.09.007

[ref37] Kret M. De Gelder B. (2013). When a smile becomes a fist: the perception of facial and bodily expressions of emotion in violent offenders. Exp. Brain Res. 228, 399–410. doi: 10.1007/s00221-013-3557-6, PMID: 23828232 PMC3710410

[ref38] Lila M. Martín-Fernández M. Gracia E. López-Ossorio J. J. González J. L. (2019). Identifying key predictors of recidivism among offenders attending a batterer intervention program: a survival analysis. Psychosoc. Interv. 28, 157–167. doi: 10.5093/pi2019a19

[ref39] Madary M. Metzinger T. K. (2016). Recommendations for good scientific practice and the consumers of VR-technology. Front. Robot. AI 3:3. doi: 10.3389/frobt.2016.00003

[ref40] Mandler G. Mandler J. M. Uviller E. T. (1958). Autonomic feedback: the perception of autonomic activity. J. Abnorm. Soc. Psychol. 56:367. doi: 10.1037/h0048083, PMID: 13538604

[ref41] Mann R. E. Barnett G. D. (2013). Victim empathy intervention with sexual offenders: rehabilitation, punishment, or correctional quackery? Sex. Abus. 25, 282–301. doi: 10.1177/1079063212455669, PMID: 22915205

[ref42] Mariano M. Pino M. C. Peretti S. Valenti M. Mazza M. (2017). Understanding criminal behavior: empathic impairment in criminal offenders. Soc. Neurosci. 12, 379–385. doi: 10.1080/17470919.2016.1179670, PMID: 27108546

[ref43] Marloth M. Chandler J. Vogeley K. (2020). Psychiatric interventions in virtual reality: why we need an ethical framework. Cambridge Q. ofHealthcare Ethics 29, 574–584. doi: 10.1017/S0963180120000328, PMID: 32892774

[ref44] Marsh A. A. Blair R. J. R. (2008). Deficits in facial affect recognition among antisocial populations: a meta-analysis. Neurosci. Biobehav. Rev. 32, 454–465. doi: 10.1016/j.neubiorev.2007.08.003, PMID: 17915324 PMC2255599

[ref45] Marshall A. D. (2005). Violent husbands’ recognition of emotional expressions among the faces of strangers and their wives. PhD thesis, Indiana University, 3162247.

[ref46] Marshall A. D. Holtzworth-Munroe A. (2010). Recognition of wives’ emotional expressions: a mechanism in the relationship between psychopathology and intimate partner violence perpetration. J. Fam. Psychol. 24, 21–30. doi: 10.1037/a0017952, PMID: 20175605 PMC2886149

[ref47] Meeren H. Van Heijnsbergen C. De Gelder B. (2005). Rapid perceptual integration of facial expression and emotional body language. Proc. Natl. Acad. Sci. U. S. A. 102, 16518–16523. doi: 10.1073/pnas.0507650102, PMID: 16260734 PMC1283446

[ref48] Michalski J. H. (2017). Status hierarchies and hegemonic masculinity: a general theory of prison violence. Br. J. Criminol. 57:azv098. doi: 10.1093/bjc/azv098

[ref49] Miller S. L. Iovanni L. (2013). Using restorative justice for gendered violence. Fem. Criminol. 8, 247–268. doi: 10.1177/1557085113490781, PMID: 32762283

[ref50] Mills J. F. Loza W. Kroner D. G. (2003). Predictive validity despite social desirability: evidence for the robustness of self-report among offenders. Crim. Behav. Ment. Health 13, 140–150. doi: 10.1002/cbm.536, PMID: 14624266

[ref51] Murphy C. M. Meis L. A. (2008). Individual treatment of intimate partner violence perpetrators. Violence Vict. 23, 173–186. doi: 10.1891/0886-6708.23.2.173, PMID: 18624088

[ref52] Parsons T. D. (2015). Virtual reality for enhanced ecological validity and experimental control in the clinical, affective and social neurosciences. Front. Hum. Neurosci. 9:660. doi: 10.3389/fnhum.2015.00660, PMID: 26696869 PMC4675850

[ref53] Pence E. Paymar M. (1993). Education Groups for Men Who Batter. New York: Springer Publishing Company.

[ref54] Pereira A. Fernández R. A. Pérez M. N. (2016). “Evaluación del papel de la prisionización en la adaptación y afrontamiento en penados,” in Avances en psicología jurídica y forense. eds. Andrés A. Fariña F. Novo M. Seijo D. (Santiago de Compostela: Sociedad Española de Psicología Jurídica y Forense), 153–161.

[ref55] Polaschek D. L. L. Collie R. M. (2004). Rehabilitating serious violent adult offenders: an empirical and theoretical stocktake. Psychol. Crime Law 10, 321–334. doi: 10.1080/10683160410001662807

[ref56] Radatz D. L. Wright E. M. (2016). Integrating the principles of effective intervention into batterer intervention programming: the case for moving toward more evidence-based programming. Trauma Violence Abuse 17, 72–87. doi: 10.1177/1524838014566695, PMID: 25573844

[ref57] Rovira A. Swapp D. Spanlang B. Slater M. (2009). The use of virtual reality in the study of people’s responses to violent incidents. Front. Behav. Neurosci. 3:59. doi: 10.3389/neuro.08.059.2009, PMID: 20076762 PMC2802544

[ref58] Sanchez-Vives M. V. Slater M. (2005). From presence to consciousness through virtual reality. Nat. Rev. Neurosci. 6, 332–339. doi: 10.1038/nrn1651, PMID: 15803164

[ref59] Sanz Fernández J. Navarro Montes R. Fausor de Castro R. Altungy P. Gesteira Santos C. Morán Rodríguez N.. (2018). La escala de deseabilidad social de Marlowe-Crowne como instrumento para la medida de la deseabilidad social, la sinceridad y otros constructos relacionados en psicología legal y forense. Piscopatología Clínica Leg. y Forense 18, 112–133.

[ref60] Seidel E. M. Pfabigan D. M. Keckeis K. Wucherer A. M. Jahn T. Lamm C.. (2013). Empathic competencies in violent offenders. Psychiatry Res. 210, 1168–1175. doi: 10.1016/j.psychres.2013.08.027, PMID: 24035702 PMC3898494

[ref61] Seinfeld S. Arroyo-Palacios J. Iruretagoyena G. Hortensius R. Zapata L. Borland D.. (2018). Offenders become the victim in virtual reality: impact of changing perspective in domestic violence. Sci. Rep. 8:2692. doi: 10.1038/s41598-018-19987-7, PMID: 29426819 PMC5807352

[ref62] Seinfeld S. Zhan M. Poyo-Solanas M. Barsuola G. Vaessen M. Slater M.. (2021). Being the victim of virtual abuse changes default mode network responses to emotional expressions. Cortex 135, 268–284. doi: 10.1016/j.cortex.2020.11.018, PMID: 33418321

[ref63] Shorey R. C. Ninnemann A. Elmquist J. Labrecque L. Zucosky H. Febres J.. (2012). Arrest history and intimate partner violence perpetration in a sample of men and women arrested for domestic violence. Int. J. Criminol. Sociol. 1, 132–140. doi: 10.6000/1929-4409.2012.01.13, PMID: 25379068 PMC4219576

[ref64] Slater M. (2009). Place illusion and plausibility can lead to realistic behaviour in immersive virtual environments. Philos. Trans. R. Soc. B Biol. Sci. 364, 3549–3557. doi: 10.1098/rstb.2009.0138, PMID: 19884149 PMC2781884

[ref66] Slater M. Gonzalez-Liencres C. Haggard P. Vinkers C. Gregory-Clarke R. Jelley S.. (2020). The ethics of realism in virtual and augmented reality. Front. Virtual Real. 1:1. doi: 10.3389/frvir.2020.00001

[ref67] Slater M. Rovira A. Southern R. Swapp D. Zhang J. J. Campbell C.. (2013). Bystander responses to a violent incident in an immersive virtual environment. PLoS One 8:e52766. doi: 10.1371/journal.pone.0052766, PMID: 23300991 PMC3534695

[ref68] Slater M. Sanchez-Vives M. V. (2016). Enhancing our lives with immersive virtual reality. Front. Robot. AI 3:74. doi: 10.3389/frobt.2016.00074

[ref69] Stover C. S. Meadows A. L. Kaufman J. (2009). Interventions for intimate partner violence: review and implications for evidence-based practice. Prof. Psychol. Res. Pract. 40, 223–233. doi: 10.1037/a0012718, PMID: 34059126

[ref70] Sygel K. Wallinius M. (2021). Immersive virtual reality simulation in forensic psychiatry and adjacent clinical fields: a review of current assessment and treatment methods for practitioners. Front. Psychol. 12:673089. doi: 10.3389/fpsyt.2021.673089, PMID: 34122189 PMC8193033

[ref71] Ticknor B. (2018). Using virtual reality to treat offenders: an examination. Int. J. Crim. Justice Sci. 13, 316–325. doi: 10.5281/zenodo.2654383

[ref72] Ticknor B. Tillinghast S. (2011). Virtual reality and the criminal justice system: new possibilities for research, training, and rehabilitation. J. Virtual Worlds Res. 4:4. doi: 10.4101/jvwr.v4i2.2071

[ref73] Van Langen M. A. M. Wissink I. B. Van Vugt E. S. Van der Stouwe T. Stams G. J. J. M. (2014). The relation between empathy and offending: a meta-analysis. Aggress. Violent Behav. 19, 179–189. doi: 10.1016/j.avb.2014.02.003

[ref74] Veling W. Moritz S. Van Der Gaag M. (2014). Brave new worlds—review and update on virtual reality assessment and treatment in psychosis. Schizophr. Bull. 40, 1194–1197. doi: 10.1093/schbul/sbu125, PMID: 25193975 PMC4193729

[ref75] Wexler D. (2020). The STOP Domestic Violence Program: Group Leader’s Manual. New York: WW Norton and Co.

